# Differential expression and function of breast regression protein 39 (BRP-39) in murine models of subacute cigarette smoke exposure and allergic airway inflammation

**DOI:** 10.1186/1465-9921-12-39

**Published:** 2011-04-07

**Authors:** Jake K Nikota, Fernando M Botelho, Carla MT Bauer, Manel Jordana, Anthony J Coyle, Alison A Humbles, Martin R Stampfli

**Affiliations:** 1 Medical Sciences Graduate Program, McMaster University, Hamilton, ON, Canada; 2 Department of Pathology and Molecular Medicine, Centre for Gene Therapeutics, McMaster University, Hamilton, Ontario, Canada; 3 MedImmune LLC, Gaithersburg, MD, USA; 4 Pfizer, Cambridge, MA USA; 5 Department of Medicine, McMaster University, Hamilton, Ontario, Canada, L8N 3Z5

## Abstract

**Background:**

While the presence of the chitinase-like molecule YKL40 has been reported in COPD and asthma, its relevance to inflammatory processes elicited by cigarette smoke and common environmental allergens, such as house dust mite (HDM), is not well understood. The objective of the current study was to assess expression and function of BRP-39, the murine equivalent of YKL40 in a murine model of cigarette smoke-induced inflammation and contrast expression and function to a model of HDM-induced allergic airway inflammation.

**Methods:**

CD1, C57BL/6, and BALB/c mice were room air- or cigarette smoke-exposed for 4 days in a whole-body exposure system. In separate experiments, BALB/c mice were challenged with HDM extract once a day for 10 days. BRP-39 was assessed by ELISA and immunohistochemistry. IL-13, IL-1R1, IL-18, and BRP-39 knock out (KO) mice were utilized to assess the mechanism and relevance of BRP-39 in cigarette smoke- and HDM-induced airway inflammation.

**Results:**

Cigarette smoke exposure elicited a robust induction of BRP-39 but not the catalytically active chitinase, AMCase, in lung epithelial cells and alveolar macrophages of all mouse strains tested. Both BRP-39 and AMCase were increased in lung tissue after HDM exposure. Examining smoke-exposed IL-1R1, IL-18, and IL-13 deficient mice, BRP-39 induction was found to be IL-1 and not IL-18 or IL-13 dependent, while induction of BRP-39 by HDM was independent of IL-1 and IL-13. Despite the importance of BRP-39 in cellular inflammation in HDM-induced airway inflammation, BRP-39 was found to be redundant for cigarette smoke-induced airway inflammation and the adjuvant properties of cigarette smoke.

**Conclusions:**

These data highlight the contrast between the importance of BRP-39 in HDM- and cigarette smoke-induced inflammation. While functionally important in HDM-induced inflammation, BRP-39 is a biomarker of cigarette smoke induced inflammation which is the byproduct of an IL-1 inflammatory pathway.

## Background

Chronic obstructive pulmonary disease (COPD) is a leading cause of morbidity and mortality worldwide [1,2]. COPD is characterized as airflow limitation that is not fully reversible, progressive in nature, and associated with an abnormal inflammatory response in the lung to noxious particles or gases such as those contained within cigarette smoke [3]. The cellular components of this inflammatory response are characteristically macrophages, neutrophils, and CD8+ T lymphocytes [4-9]. A number of mediators released by these cells likely play a critical role in airflow obstruction because of their potential to induce mucus hypersecretion and alveolar destruction. Although recent studies have implicated members of the IL-1 family of cytokines in the inflammatory pathways activated by cigarette smoke [10,11], much ambiguity remains. Understanding the cellular and molecular mechanisms of cigarette smoke induced inflammation will shed light on disease pathogenesis and identify future therapeutic targets.

It is well understood that family-18 glycosyl hydrolases such as the chitinase-like molecule YKL-40 and the murine homologue breast regression protein (BRP)-39 are upregulated in a variety of inflammatory conditions [12-14]. Two members of this family of enzymatically active and inactive chitinases, acidic mammalian chitinase (AMCase) and BRP-39 have been shown to be crucial in murine models of allergic inflammation. Specifically, BRP-39 and AMCase have been shown to be a requirement for allergic sensitization in ovalbumin (OVA) and house dust mite (HDM) models of allergic airways disease [15,16]. Additionally, YKL-40 was found to be significantly elevated in smokers without COPD and further elevated in smokers with diagnosed COPD [17,18]. Moreover, human macrophages stimulated with YKL-40 produced the neutrophil chemoattractant IL-8, providing evidence that chitinases such as BRP-39 may contribute to the inflammatory response elicited by cigarette smoke. Studies in animal models, however, are needed to investigate the functional relevance and mechanism of induction of chitinases in distinct pulmonary inflammatory diseases. In murine models, cigarette smoke causes neutrophil infiltration into the lungs similar to smoke-induced inflammation in humans [19-22]. Thus, murine models may be utilized to investigate the importance of BRP-39 in cigarette smoke-induced inflammatory processes relative to the already established importance of BRP-39 in models of allergic airway disease.

In this study we sought to determine the relevance of BRP-39, in the inflammatory response elicited by cigarette smoke and house dust mite. We identify BRP-39 as a biomarker, but not a mediator, of subacute cigarette smoke-induced inflammation and identify IL-1R1 mediated pathways as critical for the induction of BRP-39. In contrast, BRP-39 was required for the expression of allergic airway inflammation. Our study shows a differential requirement for BRP-39 in cigarette smoke-induced inflammation and models of allergic asthma.

## Methods

### Animals

Female inbred C57BL/6, BALB/c mice and outbred CD1 mice (6-8 wk old) were purchased from Charles River Laboratories (Montreal, PQ, Canada). BRP-39 deficient mice, developed on a BALB/c background, and their wild type (WT) littermates were bred at Medimmune LLC, Gaithersburg, MD, USA. IL-13 deficient mice on a BALB/c background (kindly provided by A McKenzie, MRC lab, Cambridge England [23]) were bred at McMaster University. IL-1R1 knock out (KO) and IL-18 KO mice on a C57BL/6 background were obtained from The Jackson Laboratories (Bar Harbour ME, USA). All mice were maintained under specific pathogen-free conditions in an access-restricted area, on a 12-h light-dark cycle, with food and water provided *ad libitum*. The Animal Research Ethics Board of McMaster University approved all experiments.

### Cigarette smoke exposure protocol

C57BL/6, BALB/c, and CD1 mice as well as IL-13, IL-18, IL-1R1, and BRP-39 KO mice were exposed to cigarette smoke using a whole body smoke exposure system (SIU-48, Promech Lab AB (Vintrie, Sweden)) as described in detail previously [19]. Mice were exposed to 12 2R4F reference cigarettes with filters removed (Tobacco and Health Research Institute, University of Kentucky, Lexington, KY, USA) for a period of approximately 50 minutes, twice daily, for four days. This exposure period followed an initial acclimatization period whereby mice were accustomed to smoke exposure chamber over a three-day period. Control animals were exposed to room air only.

### HDM exposure protocol

WT C57BL/6 and BALB/c mice as well as IL-13, IL-1R1, and BRP-39 KO mice were exposed to HDM using a protocol that was described in detail previously [24]. Briefly, animals were anesthetized with isoflurane (Abbott Laboratories, Saint-Laurent, Quebec, Canada) using a rodent anesthetic machine (Penlon Limited Abingdon, England) and inoculated intranasally with 25 μg of HDM extract (Greer Laboratories, Lenoir, NC, USA) in 10 μl of saline, 5 days/week for two consecutive weeks.

### OVA Challenge Protocol

WT BALB/c and BRP-39 KO mice were placed into a plexiglass chamber and exposed to 1% (w/v) OVA (Grade V, Sigma-Aldrich, Oakville, ON, Canada) in sterile saline for 20 minutes daily as described previously [25]. The aerosol was generated using a Bennet twin nebulizer at a flow rate of 10 L/min. Exposure to OVA occurred after the second of the two daily cigarette smoke exposures. Two weeks of smoke exposure were utilized when establishing OVA sensitization. For the *in vivo *recall challenge, mice were exposed to aerosolized OVA for 20 minutes on three consecutive days.

### Collection of specimens

Mice were anesthetized with isoflurane and euthanized by exsanguination prior to excision of the lungs. The trachea was cannulated with a polyethylene tube (Becton Dickinson, Sparks, MD). Prior to BAL, the right lobe of the lung was tied off and placed in ice cold PBS for generating homogenates or preparing lung single cell suspensions. Bronchoalveolar lavage (BAL) fluid was collected after instilling the left lungs with 0.25 ml of ice cold 1x phosphate-buffered saline (1x PBS), followed by 0.2 ml of 1x PBS (6). Total cell numbers were counted using a haemocytometer. Cytospins were stained with Hema 3 (Biochemical Sciences Inc., Swedesboro, New Jersey, USA). 500 cells were counted per cytospin to identify mononuclear cells, neutrophils, and eosinophils. Following BAL, lungs were fixed at 30 cm H_2_0 pressure in 10% formalin for histological assessment.

### Chitinase ELISAs

Lungs were homogenized in 1 mL PBS using a Polytron PT 2100 homogenizer (Kinematica, Switzerland). AMCase and BRP-39 levels were assessed by enzyme linked immune-sorbent assay (ELISA). The assay utilized anti-BRP-39 or anti-AMCase monoclonal antibodies for capture and respective biotinylated polyclonal antibodies for development (Medimmune LLC). Streptavidin conjugated horse radish peroxidase (HRP) (R&D Systems, Mineapolis, MN) and tetramethylbenzidine (BioFX Laboratories Owings Mills, MD) provided the enzymatic reaction and 2 fold dilutions beginning at 1000 ng and 100 ng of recombinant AMCase and BRP-39 respectively (Medimmune LLC), provided the standard for quantification. To control for variability in protein concentration between homogenate samples, Bradford assay (Bio Rad, Hercules, CA) was conducted to determine the total protein of the sample. Chitinase levels were expressed as percent of total protein.

### Immunohistochemistry

Sections (4 μm) were cut from formalin-fixed, paraffin-embedded lung tissues. Antigens were retrieved by incubating tissue sections for 45 minutes in 0.01 M citrate buffer prior to incubation for 1 hour with primary anti-BRP-39 polyclonal rabbit antibody (Medimmune LLC) diluted in UltrAb diluent (Thermo Fisher Scientific, Waltham, MA) at 7 μg/mL. Recombinant AMCase at a concentration of 1 μg/mL (Medimmune LLC) was incubated for 1 hr with the primary antibody to control for cross reactivity with the similarly structured AMCase. IHC was developed with anti-rabbit Dakocytomation HRP (Dako, Glostrup, Denmark) and counterstained in a modified Mayer's Hematoxylin solution.

### Flow cytometric analysis

Lung mononuclear cells were isolated as previously described [26]. Briefly, lungs were collected in 1x phosphate-buffered saline (PBS) and cell suspensions were generated by mechanical mincing and collagenase digestion. Debris was removed by passage through nylon mesh and cells were resuspended in 1x PBS containing 0.3% bovine serum albumin (Invitrogen, Burlington, ON, Canada) or in RPMI supplemented with 10% FBS (Sigma-Aldrich, Oakville, ON, Canada), 1% L-glutamine, and 1% penicillin/streptomycin for intracellular staining (Invitrogen, Burlington, ON, Canada). 1 × 10^6 ^lung mononuclear cells were washed once with 1x PBS/0.3% bovine serum albumin (BSA) and stained with primary antibodies directly conjugated to fluorochromes for 30 minutes at 4°C. 10^5 ^live events were acquired on an LSR II (BD Biosciences) flow cytometer and data analyzed with FlowJo analysis software (TreeStar Inc. and Standford University, Palo Alto, California). The following antibodies were used for flow cytometric analysis: FITC-conjugated anti-CD11c, PE-conjugated anti-CD11b, PE-Alexa Flour 610-conjugated anti-CD4 (Invitrogen), PE-cy5-conjugated anti-CD19, PE-cy7-conjugated anti-CD69, APC-conjugated anti-MHC class II, Alexa700-conjugated anti-Gr-1 (Invitrogen), APC-Alexa750-conjugated anti-CD8 (Invitrogen), Pacific Blue-conjugated anti-CD3. All antibodies were purchased from BD Biosciences (San Jose, California) or eBioscience (San Diego, California) unless otherwise indicated.

For intracellular flow cytometric analysis, whole lung cells were cultured for 4.5 hours in the presence of phorbol myristate acetate (PMA) and ionomycin (Sigma, St. Louis, MO, USA). Intracellular staining for cytokines was performed using BD cytofix/cytoperm and BD perm/wash reagents with GolgiStop as recommended by BD Pharmingen. Intracellular cytokine staining was performed using following antibodies: FITC-conjugated anti-T1/ST2 (MD Bioproducts), PE-conjugated anti-IL-5, PE Cy 5-conjugated anti-CD86, PE Cy 5.5-conjugated anti-CD11c, APC-conjugated anti-MHC II, Alexa Fluor 700-conjugated anti-Gr-1 (Invitrogen). All antibodies were purchased from BD Biosciences (San Jose, California) or eBioscience (San Diego, California) unless otherwise indicated. Isotype controls were utilized for each stain and are demonstrated in Additional File 1.

### Statistical analysis

Data are expressed as means ± SEMs. Statistical analysis was performed with SPSS statistical software version 17.0 (Chicago, IL, USA). Univariate General Linear Model was used to assess significance; t-tests were subsequently used for 2-group comparison. Normal distribution could not be assumed for neutrophil and eosinophil data and Mann-Whitney U tests were utilized for these comparisons. Differences were considered statistically significant when p < 0.05. All statistically significant findings were repeated and data shown are representative of 2 experiments.

## Results

### Cigarette smoke-induced inflammation and expression of chitinases and chitinase-like molecules

**Figure 1:**
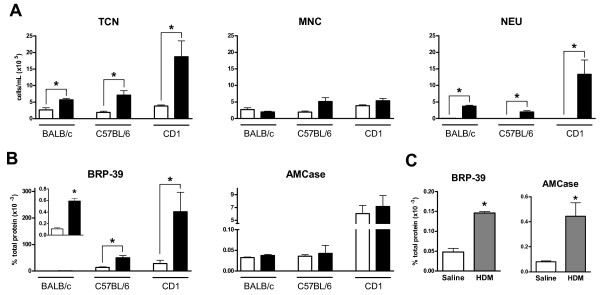
***Cigarette smoke and HDM induce chitinase expression in the lung***. BALB/c, C57BL/6, and CD1 mice were exposed to room air (white bar) or cigarette smoke (black bar) for four days. (A) Total cell numbers (TCN), mononuclear cells (MNC), and neutrophils (NEU) in the BAL fluid were obtained. (B) BRP-39 and AMCase levels were assessed by ELISA. (C) BALB/c mice were challenged with saline (white bars) or HDM (grey bars) for 2 weeks and AMCase and BRP-39 levels were assessed by ELISA in lung homogenates. n = 5, data shown are representative of two separate experiments, * indicate P < 0.05.

To investigate the impact of cigarette smoke exposure on chitinase expression, BALB/c, C57BL/6, and CD1 mice were exposed to cigarette smoke twice daily for a 4 day period. Mice were sacrificed 18 hours after their last smoke exposure. Figure 1A shows the BAL cellular profile. We observed an increased total cell number in smoke- compared to room air-exposed mice in all three strains of mice. While all of the examined strains demonstrated significantly increased numbers of neutrophils in the BAL, neutrophilia was most robust in CD1 mice and least pronounced in C57BL/6 mice.

Since chitinase expression can be induced by cigarette smoke in humans [17], we sought to measure BRP-39 and AMCase expression in lung homogenates of room air- and cigarette smoke-exposed BALB/c, C57BL/6, and CD1 mice. We observed a statistically significant increase in the chitinase-like molecule BRP-39 after smoke exposure in all mouse strains (Figure 1B). The highest baseline levels of BRP-39 and most dramatic increase in BRP-39 levels were observed in CD1 mice. In contrast to BRP-39, the enzymatically active AMCase was not increased after 4 days of smoke exposure in any of the examined mouse strains (Figure 1B). Both AMCase and BRP-39 were significantly upregulated after 2 weeks of HDM exposure (Figure 1C), confirming previous reports [15,16,27,28].

### Localization of BRP-39 expression after cigarette smoke exposure

**Figure 2:**
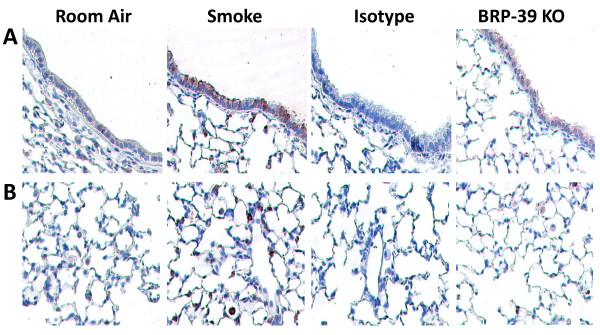
***BRP-39 is induced in lung epithelium and alveolar macrophages***. BALB/c mice were room air or cigarette smoke-exposed for 4 days. BRP-39 expression was visualized in lung tissues by immunohistochemistry. Images represent BRP-39-stained lung sections from room air and cigarette smoke-exposed mice, IgG isotype stained tissue sections from smoke-exposed mice, and BRP-39 stained tissue sections from smoke-exposed BRP-39 KO mice. Representative BRP-39 expression in (A) airway epithelium and (B) lung parenchyma are shown.

To investigate the cellular source of BRP-39 expression, we performed immunohistochemistry on formalin fixed lung tissues from cigarette smoke- and room air-exposed BALB/c mice. We observed increased BRP-39 expression in the airway epithelium following smoke exposure, although low baseline expression of BRP-39 was visible in the epithelium of room air-exposed mice (Figure 2A). Analysis of lung parenchyma revealed positive staining in alveolar macrophages in tissues from smoke-exposed mice (Figure 2B). The signal was BRP-39-specific; lung tissues from 4 day smoke-exposed mice stained with a rabbit IgG isotype control antibody and 4 day smoke-exposed BRP-39 KO mice stained with anti-BRP39 antibodies showed no signal (Representative pictures are shown in Figures 2A and 2B).

### BRP-39 induction is IL-1 dependent after subacute cigarette smoke exposure

**Figure 3:**
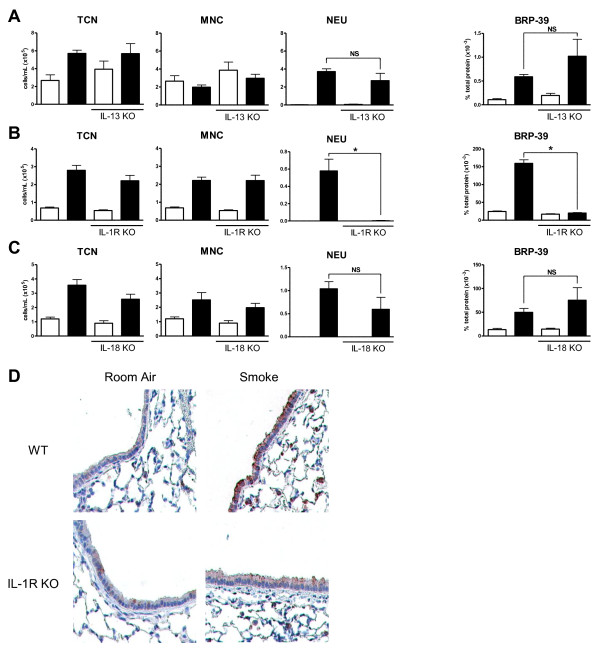
***Cigarette smoke induced BRP-39 production is IL-1 dependent***. WT BALB/c and IL-13 KO mice were room air (white bars) or cigarette smoke-exposed (black bars). (A) Data show total cell numbers (TCN), mononuclear cells (MNC), and neutrophils (NEU) in the BAL as well as BRP-39 expression in lung homogenates. WT C57BL/6 and IL-1R1 KO (B) or IL-18 KO (C) mice were room air or cigarette smoke-exposed with the same corresponding readouts. (D) Immunohistochemistry was performed to identify the localization of BRP-39 expression in WT and IL-1R1 KO mice. n = 5, data shown in B are representative of 2 separate experiments, * indicate P < 0.05.

Previous studies have implied that IL-13 is necessary to induce pulmonary BRP-39 production in models of allergic airway inflammation [15,29]. To investigate the role of IL-13 in the cigarette smoke mediated induction of BRP-39, IL-13 deficient and BALB/c control mice were smoke-exposed and BRP-39 levels were determined in lung homogenates by ELISA. Figure 3A shows that there was no difference in the cellular profile in regards to total cells, mononuclear cells, or neutrophils in the BAL as well as no difference in BRP-39 levels between smoke exposed IL-13 deficient and WT mice.

IL-1R1 and IL-18 have been shown to be crucial components in the neutrophilic inflammation elicited by cigarette smoke [10,11,30]. We therefore investigated whether IL-1R1 and IL-18 may be responsible for BRP-39 induction in this model. Mice deficient in IL-1R1, and age matched C57BL/6 mice were exposed to cigarette smoke. Analysis of BAL fluid revealed a significant attenuation of cigarette smoke induced neutrophilia in IL-1R1 KO (Figure 3B). BRP-39 expression was also abrogated in these experiments with significantly reduced BRP-39 induction in smoke exposed IL-1R1 KO mice. The same experiments were performed with IL-18 deficient and age match C57BL/6 mice (Figure 3C). Smoke-exposed IL-18 KO mice showed no significant reduction in neutrophilic inflammation when compared to smoke-exposed WT mice and no impairment in BRP-39 induction was observed. Immunohistochemistry showed a loss of BRP-39 signal in alveolar macrophages and airway epithelial cells in smoke exposed IL-1R1 KO compared to WT mice (Figure 3D). These data suggest that BRP-39 is induced by inflammatory mechanisms that are integral to the neutrophil inflammation elicited by cigarette smoke.

### HDM induced BRP-39 expression is IL-13 and IL-1 independent

**Figure 4:**
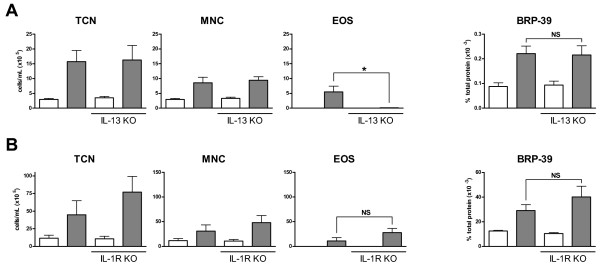
***HDM induced BRP-39 is IL-13 and IL-1 independent***. WT BALB/c and IL-13 KO mice were saline (white bars) or HDM (grey bars) exposed for 10 days. (A) Data show total cell numbers (TCN), mononuclear cells (MNC), and eosinophils (EOS) in bronchoalveolar lavage fluid as well as BRP-39 expression in lung homogenates. (B) The same readouts are shown for IL-1R1 KO mice receiving HDM exposure. n = 5-10, data shown in B are representative of 2 separate experiments, * indicate P < 0.05.

Though IL-13 is redundant to the inflammatory process and induction of BRP-39 in a model of smoke exposure, we sought to investigate whether IL-13 was essential for the induction of BRP-39 in models of allergic airway inflammation. Thus IL-13 KO and BALB/c control mice were exposed to 2 weeks of HDM. As previously reported in models of allergic airway inflammation [31], IL-13 KO mice mount a dramatically decreased eosinophilic response to HDM (Figure 4A). We observed similar expression of BRP-39 in IL-13 KO and WT control mice, inferring a redundant role for IL-13 in the induction of BRP-39 by HDM.

To determine if IL-1 is equally a critical component of BRP-39 induction in models of allergic airway inflammation, IL-1R1 KO mice were HDM exposed for a 2 week period. No significant change was observed in IL-1R1 KO mice in terms of BAL total cells, mononuclear cells, and eosinophils when compared to WT controls (Figure 4B). No detectable levels of BAL neutrophils were observed in these experiments (data not shown). Despite changes to the inflammatory phenotype, IL-1R1 KO mice demonstrated no change in BRP-39 expression (Figure 4B). Therefore, BRP-39 induction by cigarette smoke is IL-1 dependent but BRP-39 induction by HDM is IL-1 independent.

### BRP-39 is redundant in the inflammatory response to cigarette smoke

**Figure 5:**
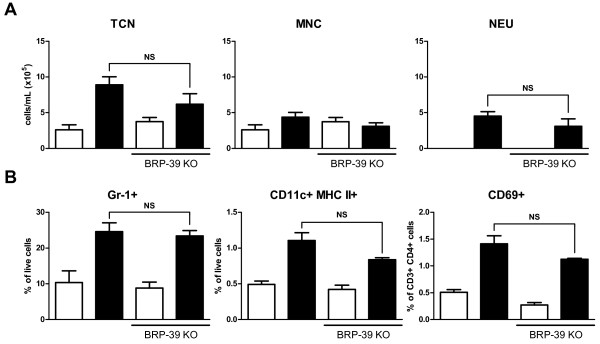
***Cigarette smoke induced inflammation is not affected by BRP-39 deficiency***. BRP-39 KO and BALB/c WT mice were room air (white bars) or smoke (black bars) exposed for four days. (A) Data show total cell numbers (TCN), mononuclear cells (MNC), and neutrophils (NEU) in BAL fluid. (B) Flow cytometric analysis of lung digests for the presence of neutrophils (Gr-1+), dendritic cells (CD11c+ MHCII+), and CD4 T cell activation (CD69+). n = 5, data shown are representative of 2 separate experiments,* indicate P < 0.05.

Having demonstrated that BRP-39 upregulation and neutrophil lung infiltration are IL-1 dependent phenomena, we sought to determine the relevance of BRP-39 to cigarette smoke-induced inflammation. BRP-39 KO mice were exposed to cigarette smoke and cellular inflammation was assessed in the BAL (Figure 5A). We observed similar total cell, mononuclear cell, and neutrophil counts in the BAL of WT and KO animals. Analysis of tissue neutrophils by flow cytometry revealed no significant differences between smoke-exposed WT and BRP-39 KO mice (Figure 5B). Previous characterization of the smoke exposure system utilized by this study confirmed an increase in dendritic cells and activation of CD4 T cells after smoke exposure [19]. Similar to tissue neutrophils, we observed no difference in dendritic cell numbers or CD4 T cell activation via flow cytometric analysis (Figure 5B). To confirm the veracity of the BRP-39 KO mice, BRP-39 expression was assessed in these mice by ELISA and no BRP-39 was detectable in the KO mice (data not shown). These data suggest that BRP-39 is redundant in the inflammatory response elicited by cigarette smoke.

### BRP-39 is not required for cigarette smoke dependent allergic sensitization

**Figure 6:**
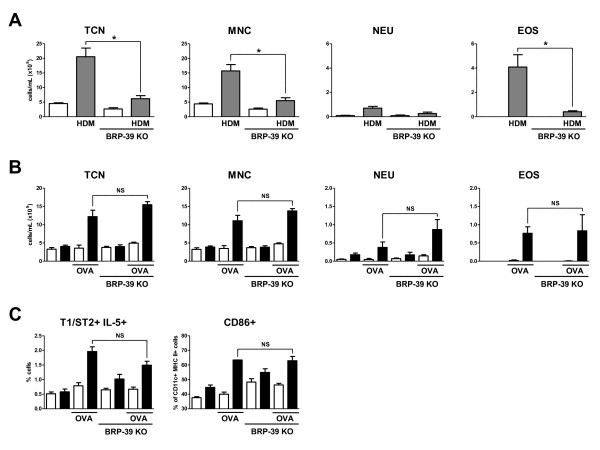
***BRP-39 is not required for cigarette smoke induced allergic sensitization***. BALB/c and BRP-39 KO mice were saline (white bars) or HDM (grey bars) exposed for 10 days. (A) Data show total cell numbers (TCN), mononuclear cells (MNC), neutrophils (NEU), and eosinophils (EOS) in bronchoalveolar lavage fluid. In separate experiments, BRP-39 KO and BALB/c WT mice were room air (white bars) or smoke (black bars) exposed for 2 weeks and concurrently exposed to nebulized OVA. Upon rechallenge following a month of smoke and OVA exposure cessation, cellular inflammation was assessed. (B) Data show total cell numbers (TCN), mononuclear cells (MNC), neutrophils (NEU), and eosinophils (EOS) in the BAL fluid. (C) Lung digests were also generated and analyzed by flow cytometry for the presence of Th2 cells (T1ST2+ IL-5+) and activated dendritic cells (CD86+). n = 5, data in B and C are representative of 2 separate experiments, * indicate P < 0.05.

Studies by Lee *et al *showed that BRP-39 plays a crucial role in processes leading to allergic sensitization to OVA and HDM [15]. To reproduce these previous findings, we exposed BALB/c and BRP-39 KO mice to HDM for 2 weeks (Figure 6A). In this model, we also observed a decrease in total cells, mononuclear cells and eosinophils in the BAL of BRP-39 KO mice when compared to their WT controls. We and others have previously reported that cigarette smoke has adjuvant properties allowing for allergic mucosal sensitization to OVA under conditions that otherwise induce inhalation tolerance [25,32]. To investigate whether BRP-39 is critical for cigarette smoke's adjuvant properties, BRP-39 KO and WT control mice were concurrently exposed to cigarette smoke and aerosolized OVA for 2 weeks. Mice were rested for 1 month prior to 3 consecutive days of OVA rechallenge. No differences were observed between BRP-39 KO mice and WT controls in terms of the BAL inflammatory profile (Figure 6B). We observed similar numbers of mononuclear cells and eosinophils in the BAL of BRP-39 and WT mice. Flow cytometric analysis of lung preparations further revealed no difference in numbers of Th2 cells (as assessed by T1/ST2 and IL-5 signal) and DC activation (as assessed by CD86+ signal on CD11c+, MHC II+ cells) between BRP-39 KO and WT mice (Figure 6C), suggesting that BRP-39 is not required for allergic sensitization in the context of cigarette smoke exposure.

## Discussion

Though the induction of BRP-39 is observed in a wide variety of inflammatory conditions and has been debated as a biomarker of certain disease states, relatively little investigation into its relevance in inflammatory responses has been made; necessitating additional study with *in vivo *models (reviewed in [33]). Thus, the objective of this study was to determine the expression and relevance of the chitinases BRP-39 and AMCase in cigarette smoke-induced airway inflammation and contrast this to HDM-induced allergic inflammation because of previously established chitinase expression in allergic airways disease.

To pursue this study, we utilized a murine whole body cigarette smoke exposure system. Mice were exposed to cigarette smoke for 4 consecutive days. This time point was chosen based on previous time course experiments to determine when a robust inflammatory response could first be reliably detected (data not shown). Though this time point is ideal for assessing cellular inflammation, the smoke exposure period is not long enough to measure lung destruction characteristic of emphysema. The inflammation induced is largely neutrophilic in nature, an observation similar to that described in COPD patients [34,35]. As further validation of this model, we previously reported levels of carboxyhemoglobin (a measurement of the saturation of hemoglobin with carbon monoxide) and cotinine (a metabolic product of nicotine) similar to the human reference [19]. Similarly, the HDM model utilized a 2 week time point as this has been previously established as the earliest time point to observe robust eosinophilic inflammation [36], while prolonged exposure is required to induce airway remodeling. Thus, the focus of both models is the inflammatory response, which is believed to drive, at least in part, the pathogenesis of COPD and asthma.

The increase in BRP-39 expression after smoke exposure is a robust event observed across inbred strains and outbred stock. This induction is in agreement with clinical observations of increased YKL-40 expression levels in smokers and COPD patients. Unlike models of allergic airway inflammation where both AMCase and BRP-39 have been shown to be elevated [15,16], increased expression levels of AMCase were not observed following smoke exposure, thus distinguishing the chitinase expression profile elicited by cigarette smoke from the one elicited by allergens.

The induction of BRP-39 and the infiltration of cells into the lungs were concurrent phenomena after 4 days of cigarette smoke exposure. IHC on lung sections implicated epithelial cells and macrophages as the primary producers of BRP-39 in this model, which is in agreement with the YKL40 expression pattern in humans and other smoke exposure models [17,18]. Others have found that neutrophils are capable of producing YKL-40 in humans [37]; however, no evidence in our model suggests that this prominent inflammatory cell type is contributing to BRP-39 production. Regardless of the relevance of BRP-39 in disease pathology, its expression is closely associated with the inflammatory response and BRP-39 remains a biomarker of inflammatory disease.

Following the initial observation of BRP-39 induction in allergic disease, Th2 mechanisms were postulated as being responsible for driving this process [15,29]. Th2 responses are believed to be crucial for parasitic defense and the induction of enzymes with the potential to break down the protective sheaths of parasitic nematodes would be of great efficacy to such responses. The finding that enzymatically active AMCase is induced in an IL-13 dependent manner in Th2 driven inflammation reinforced this hypothesis [16]. Though Th2 cytokines, including IL-13, have been detected in the smoke exposure model utilized in this study [19], IL-13 KO mice revealed that BRP-39 induction by cigarette smoke is IL-13 independent. This is not entirely surprising as IL-13 does not appear to be a critical mediator of inflammation in the smoke exposure system for its deficiency also has no effect on cellular inflammation. Conversely, it was rather unexpected that in HDM-induced allergic inflammation; which is Th2-driven, IL-13 was unnecessary for the induction of BRP-39; in other words BRP-39 induction was unaltered and yet eosinophilic inflammation was markedly attenuated. These results are at variance with previous work that implicated BRP-39 as a crucial inflammatory component in similar HDM models [15]. This represents a significant finding and expands on previous work by Lee *et al *in which IL-13 dependence for BRP-39 induction in allergic airway inflammation was strongly implied by experiments where transgenic amounts of IL-13 had been over-expressed in the lungs [15]. The experiments by Lee et al, however, did not utilize an IL-13 KO strain and as such these data only demonstrate that IL-13 is able to induce BRP-39 and not whether IL-13 is essential for BRP-39 induction. Our data show that although IL-13 is capable of inducing BRP-39 expression, it is redundant in models of cigarette smoke- and allergen-induced airway inflammation in the induction of BRP-39.

IL-1 has been implicated *in vitro *in BRP-39 induction [38]. The IL-1R1 KO mice were chosen for this reason and because IL-1R1 deficiency was sufficient to attenuate smoke-induced neutrophilic inflammation. The observation that smoke-exposed IL-1R1 KO mice did not up-regulate expression of BRP-39 suggests a crucial role of IL-1 in this phenomenon. This provides further evidence that the induction of BRP-39 is closely tied to inflammatory pathways. Further investigation of the importance of IL-1 in the induction of BRP-39 in allergic inflammation revealed that IL-1R1 was not crucial in the HDM model, highlighting the different inflammatory pathways engaged by these two models. Our data which confirms the importance of BRP-39 in HDM-induced inflammation imply that BRP-39, in the context of allergy, is part of an immune inflammatory pathway crucial to mononuclear cell and eosinophil recruitment that is not dependent on IL-1 or IL-13.

Recently Matsuura *et al *have implicated IL-18 as a mechanistic component of BRP-39 induction in a murine model of smoke exposure [18]. These data complement previous experiments that implicate IL-18 as a crucial component of cigarette smoke-induced inflammation [10]. Our data generated in IL-18 KO mice suggest that IL-18 is redundant in the inflammatory response and in the induction of BRP-39 which was confirmed by experiments with IL-18 receptor KO mice (data not shown). This discrepancy could be the result of different smoke exposure conditions as Matsuura *et al *utilized a nose only smoke exposure apparatus characterized by Shapiro *et al *[39], as opposed to a whole body smoke exposure system. A more likely explanation of the discrepancy is the length of smoke-exposure, as our study exposed mice to smoke for four days while Matsuura *et al *exposed mice to smoke for a month to determine the mechanistic relevance of IL-18. The four day time point was chosen for this study because experiments showed a greater induction of BRP-39 at subacute time points when compared to the chronic setting (data not shown). These findings taken in context with the data from IL-1R1 KO mice imply a timeline for cigarette smoke induced inflammation where IL-1 inflammatory pathways are more important early on in disease progression with IL-18 mediated pathways engaged after sustain cigarette smoke stimuli.

Evidence such as the stimulation of cells with YKL-40 inducing inflammatory chemokines has implied a role for this YKL-40 and BRP-39 in cellular inflammation [17,38], yet BRP-39 deficiency did not lead to significantly attenuated lung-infiltrating cell types after smoke exposure. The redundant nature of BRP-39 in this inflammatory response represents the most striking finding of this study and again contrasts the work by Matsuura *et al *[18]. As stated before, this is likely the result of the different durations of smoke exposure as Matsuura *et al *did not witness reduced inflammation in smoke-exposed BRP-39 KO mice until at least 3 months of smoke-exposure. This implicates BRP-39 in the survival of inflammatory cells in a chronic inflammatory setting and not in the initial recruitment of cells to the lungs. The lack of significant difference in tissue neutrophils, DCs, and CD4 T cell activation more specifically reinforces the redundant nature of BRP-39 in the early stages of cigarette smoke-induced inflammation.

Another striking conclusion of these experiments was that although BRP-39 has been shown to be crucial for allergic sensitization, it is redundant in the adjuvant properties of cigarette smoke. This implies a different mechanism of sensitization when cigarette smoke is utilized as an adjuvant. This is not an unprecedented assertion as HDM models of allergic sensitization and models of cigarette smoke induced OVA sensitization have been shown to utilize different inflammatory pathways [40]. Lee *et al *postulated that the attenuation of allergic responses in BRP-39 deficient mice was due to an increase in apoptosis of a key mediating cell type [15]. Apoptosis was not assessed in this study but if there was increased apoptosis in BRP-39 deficient animals it was not sufficient to impede sensitization or decrease the amount of activated DCs, implying that an increase in apoptosis may not be sufficient to interrupt sensitization when alternate pathways are driving sensitization. This is likely the case when cigarette smoke is utilized as an adjuvant.

## Conclusions

In conclusion, these results demonstrate that BRP-39 is a biomarker of cigarette smoke- and allergen-induced inflammation. Its induction by cigarette smoke is IL-1R1 dependent, which is unique from BRP-39 induction in HDM-induced allergic inflammation which is both IL-1R1 and IL-13 independent. Despite the fact that BRP-39 is induced by an inflammatory agent, BRP-39 is itself redundant in cigarette smoke-induced inflammation. Also, despite being a crucial mediator of allergic sensitization in widely utilized models of airway inflammation, BRP-39 is not crucial for the adjuvant properties of cigarette smoke. This study highlights the inflammatory mechanism elicited by cigarette smoke to induce BRP-39 expression which is unique from allergic inflammation as well as the function of BRP-39 in subacute smoke exposure and cigarette smoke induced allergic sensitization.

## Competing interests

The authors declare that they have no competing interests.

## Authors' contributions

JKN conducted mouse experiments, aided in experiment design, performed IHC and ELISAs, participated in flow cytometric analysis and drafted the manuscript. FMB participated in mouse experiments and conducted flow cytometry. CMTB participated in mouse experiments and manuscript preparation. MJ, AJC, and AAH participated in the design of the study helped to draft the manuscript. MRS conceived and designed the study and aided in drafting the manuscript. All authors read and approved the manuscript.

## Supplementary Material

Additional File 1 ***Isotype controls for flow cytometry data***. The appropriate isotype controls are shown in flow cytometry pseudo-dot plots of data generated from for the lung digests of 4 day smoke exposed lungs (A,C,D) and smoke- and OVA-exposed mice after 1 month of cessation and 3 days of rechallenge with OVA (B,E). Histogram data (C-E) contrasts positive stain (black line) with the appropriate isotype control (solid grey line). Click here for file
