# Supplementary material for: Differential expression and function of breast regression protein 39 (BRP-39) in murine models of subacute cigarette smoke exposure and allergic airway inflammation
Source: Respir Res. 2011 Apr 7;12(1):39. doi: 10.1186/1465-9921-12-39 (PMC3079621; doi:10.1186/1465-9921-12-39)
Supplement: Additional File 1 — Isotype controls for flow cytometry data. The appropriate isotype controls are shown in flow cytometry pseudo-dot plots of data generated from for the lung digests of 4 day smoke exposed lungs (A,C,D) and smoke- and OVA-exposed mice after 1 month of cessation and 3 days of rechallenge with OVA (B,E). Histogram data (C-E) contrasts positive stain (black line) with the appropriate isotype control (solid grey line). [file 1465-9921-12-39-S1.PDF]

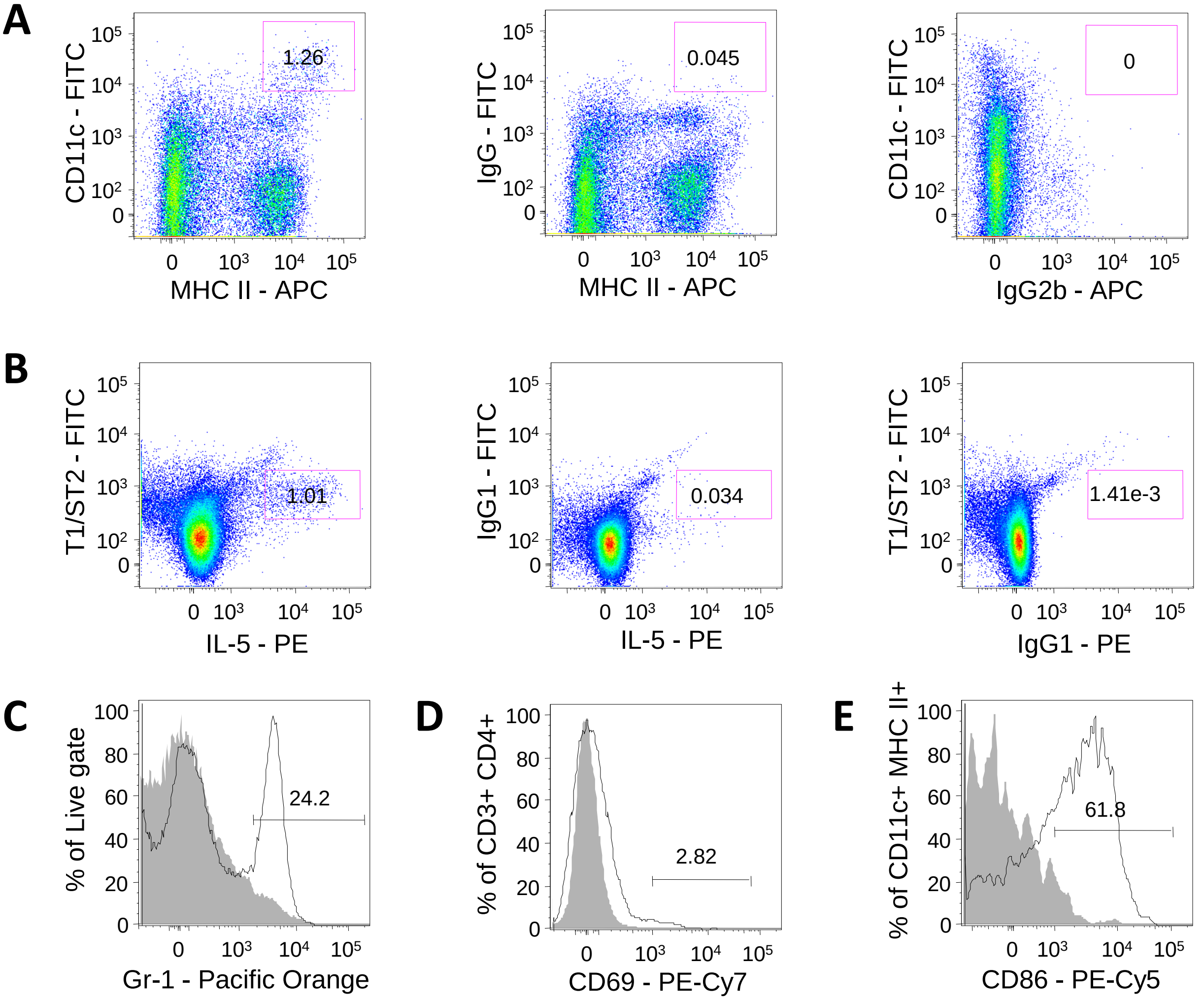

Additional File 1 *Isotype controls for flow cytometry data*. The appropriate isotype controls are shown in flow cytometry pseudo-dot plots of data generated from for the lung digests of 4 day smoke exposed lungs (A,C,D) and smoke- and OVA-exposed mice after 1 month of cessation and 3 days of rechallenge with OVA (B,E). Histogram data (C-E) contrasts positive stain (black line) with the appropriate isotype control (solid grey line).
